# Coupled Effect of Polypropylene Fibers and Slag on the Impact Resistance and Mechanical Properties of Concrete

**DOI:** 10.3390/ma15165654

**Published:** 2022-08-17

**Authors:** Abdul Basit Ali, Muhammad Burhan Sharif, Muhammad Irfan-ul-Hassan, Yasir Iqbal, Usman Akmal, Hisham Alabduljabbar, Ahmed Farouk Deifalla

**Affiliations:** 1 Department of Civil Engineering, University of Engineering and Technology, Lahore 54890, Pakistan; 2 Department of Civil Engineering, College of Engineering in Al-Kharj, Prince Sattam Bin Abdulaziz University, Al-Kharj 11942, Saudi Arabia; 3 Structural Engineering Department, Faculty of Engineering and Technology, Future University in Egypt, New Cairo 11835, Egypt

**Keywords:** steel slag, impact resistance, sand replacement, polypropylene fibers, compressive strength

## Abstract

The disposal of steel slag leads to the occupation of large land areas, along with many environmental consequences, due to the release of poisonous substances into the water and soil. The use of steel slag in concrete as a sand-replacement material can assist in reducing its impacts on the environment and can be an alternative source of fine aggregates. This is the very first paper that seeks to experimentally investigate the cumulative effect of steel slag and polypropylene fibers, particularly on the impact resistance of concrete. Various concrete mixes were devised by substituting natural sand with steel slag at volumetric replacement ratios of 0%, 10%, 20%, 30%, and 40%, with and without fibers. Polypropylene fibers of 12 mm length were introduced into the steel slag concrete at 0%, 0.5%, and 1.0% by weight of cement as a reinforcing material. Performance evaluation of each mix through extensive experimental testing indicated that the use of steel slag as partial substitution of natural sand, up to a certain optimum replacement level of 30%, considerably improved the compressive strength, flexural strength, and tensile strength of the concrete by 20.4%, 23.8%, and 17.0%, respectively. Furthermore, the addition of polypropylene fibers to the steel slag concrete played a beneficial role in the improvement of strength characteristics, particularly the flexural strength and final drop weight impact energy, which had a maximum rise of 48.1% and 164%, correspondingly. Moreover, integral structure and analytical analyses have also been performed in this study to validate the experimental findings. The results obtained encourage the use of fiber-reinforced steel slag concrete (FRSLC) as a potential impact-resistant material considering the environmental advantages, with the suggested substitution, of an addition ratio of 30% and 1.0% for steel slag and polypropylene fibers, respectively.

## 1. Introduction

The global inclination toward industrial development has made waste management one of the most perplexing dilemmas. The economic advantages of this rapid industrialization go hand in hand with the production of environmentally hazardous wastes [1]. As an illustration, the growing industry of steel production from around the globe is incredibly good for the economic growth of countries, but the generation of slag along with steel is equally bad for the environment [2]. Steel slags are non-metallically inert and mainly consist of silicates, aluminosilicates, and calcium aluminosilicates, whose production cannot be eliminated [3,4]. In the manufacturing process of iron and steel, fluxes consisting of sedimentary rock and/or dolomite are introduced into the furnace, along with coke as a fuel. Carbon monoxide is produced upon the combustion of the coke, which converts the iron ore into liquid iron, and a restricted quantity is obtained on the prime surface of the iron as a slag, comprising about 15–20% of the total iron/steel production [5,6]. Currently, the statistical data relating to China, Japan, and Europe reveal that more than 100, 21 and 14 million tons of steel slag, respectively, are being generated annually [7,8], and this value is increasing day by day. The huge quantities of slags not only occupy large land areas but also pollute the natural water and soil due to the proclamation of alkaline leachates [8,9,10]. Researchers are trying their best to find new methods which could minimize slag production [11,12]. Meanwhile, their research efforts are focused on the minimization of slag generation; the use of slags in the concrete will not only help in reducing greenhouse gases (GHG), but will also make the environment better for living [7,13,14].

Furthermore, the construction industry is looking for sustainable, economical, and environmentally friendly construction systems for saving the world. Therefore, the use of steel slag in concrete is a promising concept [15], as concrete is the second-most-consumed material after water [16,17]. Concrete is a blend of mainly cement; it is an aggregate stack combination of coarse and fine aggregates along with water, which is easily obtainable in most countries around the world, and sometimes with the addition of supplementary cementitious materials and/or chemical admixtures to minimize the various construction hazards. Nearly 60~75% of the concrete volume consists of coarse and fine aggregates [18]; thus, the use of steel slag as sand-replacement material in concrete will not only help in utilizing its large quantities and diminishing harmful effects [19], but will also lead to cost savings in materials and maintenance [7,13,17,20]. In search of finding ecological and sustainable ways of construction, few researchers have made use of steel slag as an aggregate replacement material in concrete and tested it under the impact-type loadings. Guo et al. [13] conducted the very first research study to examine the behavior of steel slag–sand concrete under high-velocity axial impact compressive loadings; the study concluded that the axial compressive strength of steel slag–sand concrete is significantly more than normal concrete, but it results in amplified stiffness and brittleness, with smash failure in the concrete specimens. Devi and Gnanavel [18] introduced the steel slag in numerous concrete mixtures as a fractional substitution for coarse and fine aggregates, and found that the use of steel slag as a fine aggregate has a more prominent effect on compressive and tensile strength than its use as a coarse aggregate. Qasrawi et al. [21,22] studied the utilization of low CaO and high Fe_2_O_3_ untreated slag in concrete as a sand-replacement material and found marginal improvements in different strength characteristics of the concrete. Furthermore, Guo et al. [14] investigated the use of unprocessed steel slag in normal- and high-strength concrete mixtures; the results indicate that the use of steel slag highly alleviates the energy absorption capacities and compressive strength of the concrete, rendering slag a suitable sand-replacement material. In addition, the masonry mortars made from steel slags have also been reported feasible by Santamaría-Vicario et al. [23]. Recently, Lai et al. [4] conducted a scientific study by replacing natural sand with steel slag and found a notable improvement in the compressive strength of the mixes. 

On the other hand, the construction industry is also concerned about the brittle nature of concrete, which has low tensile strength and behaves poorly under impact-type loadings [24,25,26], and is looking for improvements in its basic composition leading to amelioration in its energy-absorbing capacity [27,28,29]. Concrete is subjected to many types of impact loading [30], such as concrete pavements for heavy vehicles and aircrafts, concrete dividers and barriers on highways, and structural concrete subjected to explosions [12,31,32]. The impact of objects hitting the concrete at varying velocities can damage it as a whole or partially with disintegrated concrete debris that can fly [33,34,35]. Enhancement of impact resistance of the concrete has always been an uneconomical issue [36]. The methods employed to cater to this problem include increases in the cross-sectional size of members or the introduction of a heavy amount of reinforcement detailing, which ultimately results in unwanted higher project costs [37,38,39]. Conventional structural materials are not resistant enough to bear the impact of explosives [12,32]. Therefore, there is a growing necessity to introduce advanced solutions to the problem. To improve the ductility and energy absorbing capacity of concrete, the use of fibers in concrete has been reported as an auspicious solution by various researchers [28,29,40,41,42,43]. Omar and Hassan [28] conducted an experimental investigation to improve the impact resistance and mechanical properties of lightweight concrete by the use of polymeric fibers; the study revealed that polypropylene fibers greatly improved the drop weight impact resistance and ductility of the concrete, along with the flexural strength. Das et al. [39] stated that PP fibers play a vital role in the improvement of tensile and flexural strengths of concrete made up of low-strength recycled aggregates when used in limited quantities. Moreover, the role of PP fibers in the production of high-strength concrete has been reported in a study by Afroughsabet and Ozbakkaloglu [34]. Zia and Ali [40] investigated the use of three types of fibers (Jute, Nylon, and Polypropylene fibers) in the concrete for controlling the rate of cracking in canal lining. It was found that Polypropylene fibers were the most effective in controlling cracks among all other fiber types as they effectively prevented and suppressed the formation of cracks in the concrete. Furthermore, Ashraf et al. [36] probed the effect of polymer-based SBR latex combined with PP fibers on the impact behaviors of the concrete and found encouraging results. 

Previous studies revealed that few researchers have examined the use of steel slag as fine aggregate in concrete to enhance its static performance, whereas limited research data exist on the impact behaviors of the steel slag concrete. Additionally, researchers either focused on the improvement of the environment by using industrial wastes or on the brittle nature of concrete with the adaptation of fibers, not considering both aspects. In recent times, most of the construction carried out consists of concrete structures which behave poorly under impact loadings. Any improvement in the basic composition of concrete leading to amelioration in its energy absorbing capacity would have a profound effect on the construction industry. To overcome the inherent weakness of concrete structures under impact loadings, the use of polypropylene fibers in steel slag concrete can be a promising solution for enhancing the impact performance of concrete as they are abundantly available in most countries and are less costly than steel fibers. Therefore, this research aims to experimentally examine the impact resistance and mechanical properties of fibrous and non-fibrous concrete, with waste steel slag as sand-replacement material and the addition of polypropylene fibers, for the very first time. Consequently, the authors believe that the coupled effect of polypropylene fibers and waste steel slag in concrete will contribute to the efforts of the community for a sustainable environment and will also be a valuable addition to the civil engineering world by evolving energy absorbent construction materials for utility in various infrastructure projects.

## 2. Materials and Methods

This section deliberates on the material properties, concrete mix proportions, and testing procedures that were implemented to obtain the desired parameters.

### 2.1. Materials

The Ordinary Portland cement (OPC, ASTM Type I) meeting the requirements of ASTM C150/C150M-21 [44] was used in this experimental investigation. The chemical composition and other physical parameters of the cement are given in Table 1. Natural sand of Fineness modulus 2.52 in combination with the locally available waste steel slag was used as fine aggregates. In most countries, steel is produced using old steel instead of iron ores; therefore, huge quantities of waste slags rich in iron oxide content (Fe_2_O_3_) of various shapes ranging from massive boulders to dust were generated. Based on the particle size, waste slags are mainly categorized into three types comprising large boulders, sand-sized scrape, and extremely fine dust particles.

**Figure 1:**
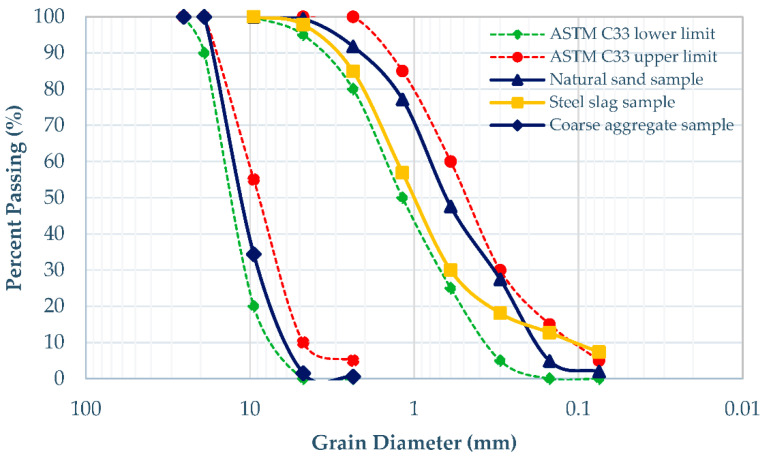
Particle size distribution curves of coarse and fine aggregates.

In the current study, sand-sized (4.75 to 0.075 mm) “Scrape” steel slag was used in concrete mixes in the “as received” form without any added screening to economize the mix designs. Physical observation showed that the fine aggregates were clean, free from clay and any other organic deleterious substances. The chemical composition of steel slag was also analyzed and has been compared with the cement in Table 1. Very low contents of CaO and SiO_2_ are evident that there are negligible chances of pozzolanic activity due to steel slag. Coarse aggregates of particle size ranging from 19.0 to 4.75 mm were employed in this research. Particle size distribution of aggregates was performed by implementing ASTM C136/C136M-14 [45], and results were compared with the gradation requirements of ASTM C33/C33M-13 [46], as shown in Figure 1. The other physical properties of aggregates are summarized in Table 2 and Table 3. The use of 12 mm polypropylene fibers, with their properties given in Table 4, was also found in experimentations to meet the research objectives. A chloride-free high-performance polymer-based superplasticizer of reddish-brown color, having specific gravity of 1.165 ± 0.005, with the local trade name of Expanplast* SP511 (Fospak), was also used to boost the low workability of the mixtures.

### 2.2. Concrete Mix Proportions

To examine the effects of natural sand substitution with steel slag and the addition of polypropylene fibers on mechanical performance and impact behavior of concrete, fifteen concrete mixes were cast in the research laboratory utilizing the materials introduced in Section 2.1. The concrete ingredient proportions were calculated as per American Concrete Institute ACI 211.1-91 [47] Mix Design guidelines for the minimum cylindrical strength of 28 MPa. The contents of mix designs are produced in Table 5. The water–cement ratio was kept constant in all mixes at 0.48. These mixes were categorized into three concrete groups based on different fiber content. Each concrete group had 0%, 10%, 20%, 30%, and 40%, steel slag as sand-replacement material. The sand replacement with steel slag was made by volume since the difference in specific gravities of sand and steel slag was significant. The specific gravity of sand being used was 2.76, whereas this value was 3.15 for steel slag. Out of the three concrete groups, no fibers were present in one group and the other two groups had polypropylene fibers at a dosage addition rate of 0.5% and 1.0% by weight of cement. The higher dosages of PP fibers were not investigated as they make the mixes unworkable, form weaker points that act as voids, and do not contribute significantly to the strength parameters [39] which were authenticated in this study later on. 

### 2.3. Casting and Curing of Specimens

Subsequently, after finding out the properties of materials and mix proportions of materials, different-shaped concrete specimens were cast to acquire the dimensions given in Table 6. Concrete ingredients were mixed in a horizontal drum mixer of 2.0 cft. (0.056 m^3^) capacity. For the preparation of slag only concrete group mixes, the cement and aggregates were mixed in a dry condition for three minutes first, then water was added. Then, the horizontal drum was further rotated for three to five minutes for uniform mixing. The desired slump range of 75–125 mm was easily achieved in normal concrete with a w/c ratio of 0.48 without requiring the use of a super-plasticizer. However, with this fixed w/c ratio, hybrid mixes having steel slag and fibers were not workable. The lower workability of mixes was compensated by adding super-plasticizer in varying dosages of 0.9~1.01% to achieve the desired slump. As far as the mixing of the other two fibrous concrete groups was concerned, the concrete ingredients were added to the mixer by a three-layer method to ensure uniform fiber dispersion [27,41]. The sequence of each layer was the coarse aggregates layer, followed by the steel slag, natural sand, polypropylene fibers, and finally, the cement layer. The mixer was revolved for three minutes after the addition of all ingredients for dry mixing, then two-thirds of the total water quantity was added. Finally, a solution of super-plasticizer and one-third of the remaining water was supplemented, and the mixer was rotated again for three to seven minutes for a homogeneous mix. 

**Figure 2:**
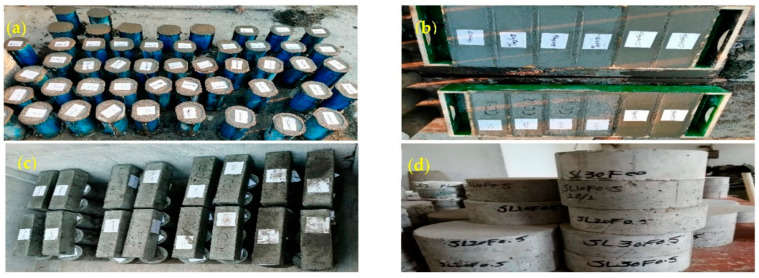
(**a**) Cast cylindrical specimens, (**b**) cast prisms for flexural strength test, (**c**) demolded prisms and cylinders, (**d**) concrete discs obtained by the cutting of cylinders for the performance of the drop weight impact test.

Three specimens for each set of test parameters were prepared. Before casting, the molds were thoroughly cleaned, bolted, and oiled from the inside. Slump tests were conducted on each concrete mix, according to ASTM C143/C143M-15a [48]. Concrete of required consistency was then filled in molds in three equal layers and compacted through tamping rods. The concrete surface was properly leveled after casting, and demolding was carried out after 24 h. Demolded specimens were stored carefully without any damage to specimens in water storage tanks for 28 days with free water retained on their surfaces at a temperature of 23 ± 2 °C [49]. Images of different-sized specimens are shown in Figure 2.

### 2.4. Test Methods

Experiments were performed for the determination of different strength parameters as per the applicable procedures illustrated in the ASTM standards listed in Table 6. However, methods adopted for the determination of compressive strength parameters, impact test, and microstructural analysis are only described in this section due to some allied accessories in the test setups.

#### 2.4.1. Method for the Determination of Compressive Strength Parameters

**Figure 3:**
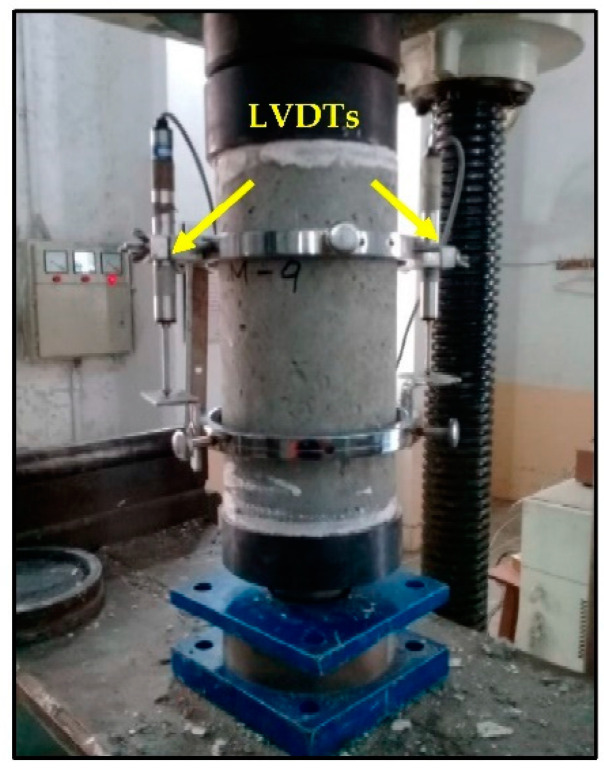
Experimental configuration for determination of compressive strength, stress–strain relationship, and modulus of elasticity.

ASTM C39/C39M-15a [50] was implemented for the performance of the compressive strength test. The compressive strength test setup is shown in Figure 3. The load-deformation data were noted down using the data acquisition system attached to the compression testing machine. Load cells along with two linear variable displacement transducers (LVDTs) were subsequently installed to trace the stress–strain curves. The compressive stress and strain values were calculated as $\sigma_{c}=P/A$, and ε=Δl/L, respectively. This test setup was carried out to ascertain compression behavior and compressive strength *(*${f_{c}}^{\prime }$*)* on samples of 150 mm diameter and 300 mm height. 

Moreover, the same setup was used for the computation of modulus of elasticity, which is the ratio of change in compressive stress $\Delta \sigma_{c}\;$to change in compressive strain ∆*ε* within the elastic limit. Stress–strain curves and Equation (1), as per guidelines of ASTM, C469/469M-14 [51] were applied in this regard.

(1) $$E_{c}=\Delta \sigma_{c}/\Delta \varepsilon =\frac{\left(\sigma_{2}-\sigma_{1}\right)}{\left(\varepsilon_{2}-0.00005\right)}$$

where: 

$\sigma_{2}$= Stress subsequent to 40% of maximum load.

$\sigma_{1}$ = Stress subsequent to longitudinal strain of 0.00005.

*ε*_2_ = Longitudinal strain produced due to $\sigma_{2}$.

#### 2.4.2. Method for the Determination of Impact Strength

**Figure 4:**
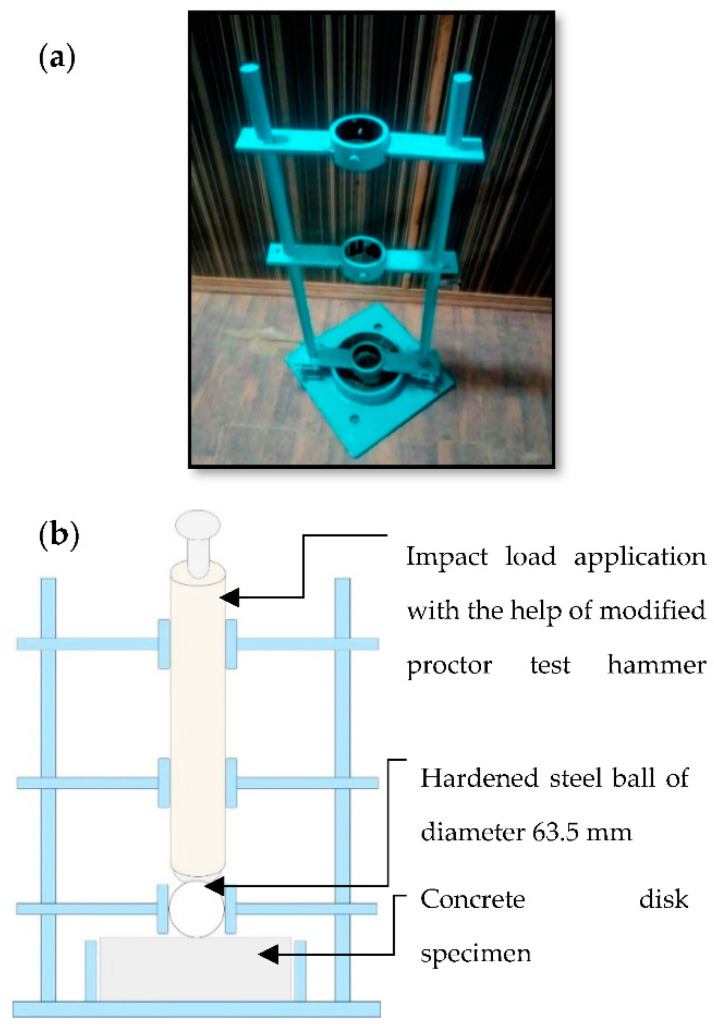
(**a**) Assembly prepared for the performance of drop weight impact test; (**b**) schematic diagram of the assembly.

The ACI committee in its report entitled ACI 544.2R-89 [52] explains various methods of testing the concrete under impact forces. The methods include the drop weight impact test, Charpy impact test, projectile impact test, explosive test, and instrumented impact tests. In this research, a drop weight impact strength test was implemented. The developed assembly for the performance of this test mainly consisted of: (1) a standard, manually operated compaction hammer with an 18-in (457 mm) drop [53], (2) a $2_{2}^{1}$″ (63.5 mm) diameter hardened steel ball, and (3) a flat base plate of $\frac{1}{4}$″ thickness with positioning brackets. The equipment and its schematic diagram are shown in Figure 4. 

The drop weight impact test was performed on the concrete disks with a diameter of 150 mm, and height of 63.5 mm. The disks were obtained by cutting the standard cylinder of 150 mm diameter into the required height of 63.5 mm. According to this method, a hammer of 4.5 kg was repeatedly dropped from a height of 457 mm on a concrete disk of 63.5 mm in height and 150 mm in diameter. The impact was transferred from the hammer to the concrete specimen through a hardened steel ball of diameter 63.5 mm. This method serves the dual purpose of uniformly transferring impact force in each hammer drop and developing an effective shock wave due to point impact loading. The steel ball was kept in its place on the concrete disk with the help of a steel pipe of a slightly larger diameter than the steel ball connected to steel plates on both sides. The steel plates were rigidly connected to the side columns of the testing apparatus. The hammer drops were continued until the first visible crack was observed on the concrete specimen surface. Once the initial crack was observed, the number of drops of the hammer to cause the initial crack was recorded. The hammer drops were again continued until the final failure of the specimen occurred. The final fracture was considered as a state in which the concrete specimen splits into several fragments and touches the boundary of the specimen. The impact resistance of fibrous and non-fibrous steel slag concrete was then determined in terms of initial and final impact energies using the counted number of drops as per Equations (2) and (3), respectively.

(2) $$E_{i}=N_{i}mgh$$

(3) $$E_{f}=N_{f}mgh$$

where:

*m* = Mass of hammer used = 4.5 kgs.

*g* = Gravitational acceleration = 9.81 ms^−2^.

*h* = Height of fall of the hammer = 457 mm.

## 3. Results and Discussion

This section presents the explanation of findings from experimental work. The mechanical performance of each concrete group has been evaluated by compressive strength, modulus of elasticity, flexural strength, splitting-tensile strength, unit weight, and water absorption test, and the results are summarized in Table 7. Interpretation of the drop weight impact test results has also been made separately. Finally, the microstructural and analytical analyses have been discussed at the end of this section.

### 3.1. Compressive Strength

**Figure 5:**
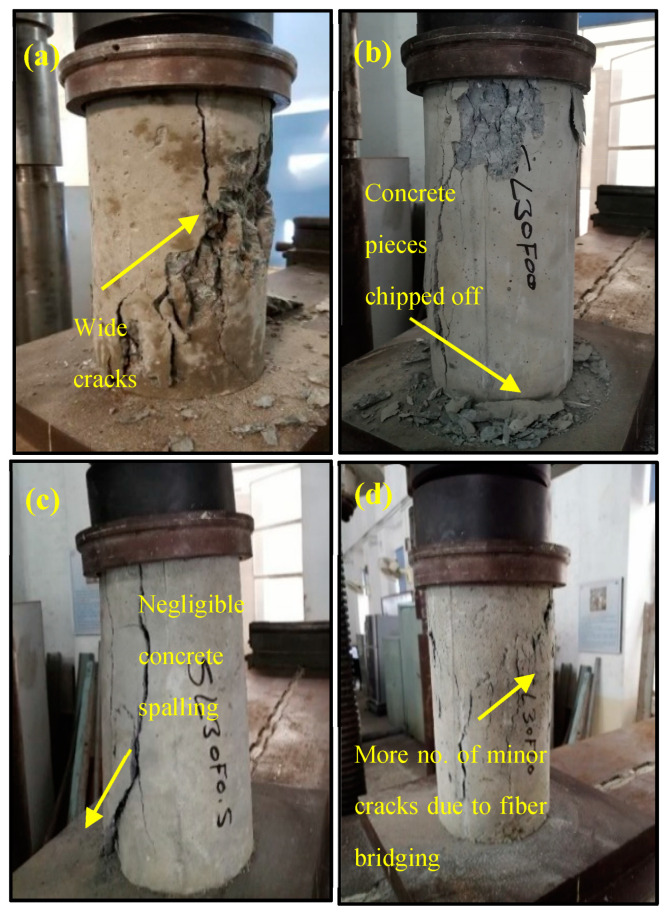
Observed failures in compression test of (**a**) control concrete, (**b**) slag + 0.0% PP fibers group, (**c**) slag + 0.5% PP fibers group, (**d**) slag + 1.0% PP fibers group.

From the comparative studies of the experimental results, reflected in Table 7, it is noted that the compressive strength of concrete surges with the increase in slag substitution ratio. The enhancement in compressive strength due to the replacement of natural sand with 10%, 20%, 30%, and 40% steel slag is 3.6%, 10.4%, 20.4%, and 6.5%, respectively. An increase in compressive strength is interrelated to the high angularity of steel slag in comparison with the natural sand, which ultimately showed a better bond with cement paste. However, the upsurge in compressive strength is not monotonous with the increase in steel slag content as compressive strength significantly decreased beyond 30% slag replacement. When slag substitution is increased further than 30%, the water demand of the mix was increased due to the surface roughness of highly angular steel slag. As the w/c ratio of all mixes was fixed to 0.48, this increased water demand was not compensated by further water addition, which led to an insufficient hydration process of cement that ultimately resulted in the loss of workability and reduction in compressive strength. A decrease in compressive strength can also be attributable to the inadequate cement quantity for effective coating of steel slag at high replacement levels of natural sand with steel slag [13]. These findings are in line with the conclusions of Qasrawi et al. [21], who observed that the increase in compressive strength is at maximum when steel slag is substituted with fine aggregates between 15% and 30%. The inclusion of polypropylene fiber in control and steel slag concrete slightly increased the compressive strength. The further increase in compressive strength was 1.0%, 1.9%, 5.1%, 4.1%, and 1.2% upon addition of 0.5% polypropylene fibers, and this increase was 3.0%, 2.9%, 5.6%, 5.1%, and −0.1% upon the addition of 1.0% polypropylene fibers, when compared with compressive strength of their respective non-fibrous specimens with 0%, 10%, 20%, 30% and 40% steel slag. This minimal effect of polypropylene fibers on the compressive strength may be due to the varied length of PP fibers in staple. Polypropylene fibers due to their bridging effect formed a network to restrict micro-cracks from propagation. Figure 5 indicates that fibrous concrete groups have much less concrete spalling than the non-fibrous concrete group. PP fibers provided confinement and increased compressive strength because of their ability to reduce stress concentration at crack tips and slow down the growth rate of cracks. These results are similar to the experimental outcomes of Aslani and Nejadi [33], who reported increased compressive strength upon the addition of polypropylene fibers in different volumetric fractions to concrete. Moreover, when fiber contents are high, these fibers may also accumulate due to improper mixing or loss of mobility and form weaker points that act as voids and therefore become more prone to cracking, thus reducing the compressive strength [39].

### 3.2. Compressive Stress–Strain Relationship and Elastic Modulus

**Figure 6:**
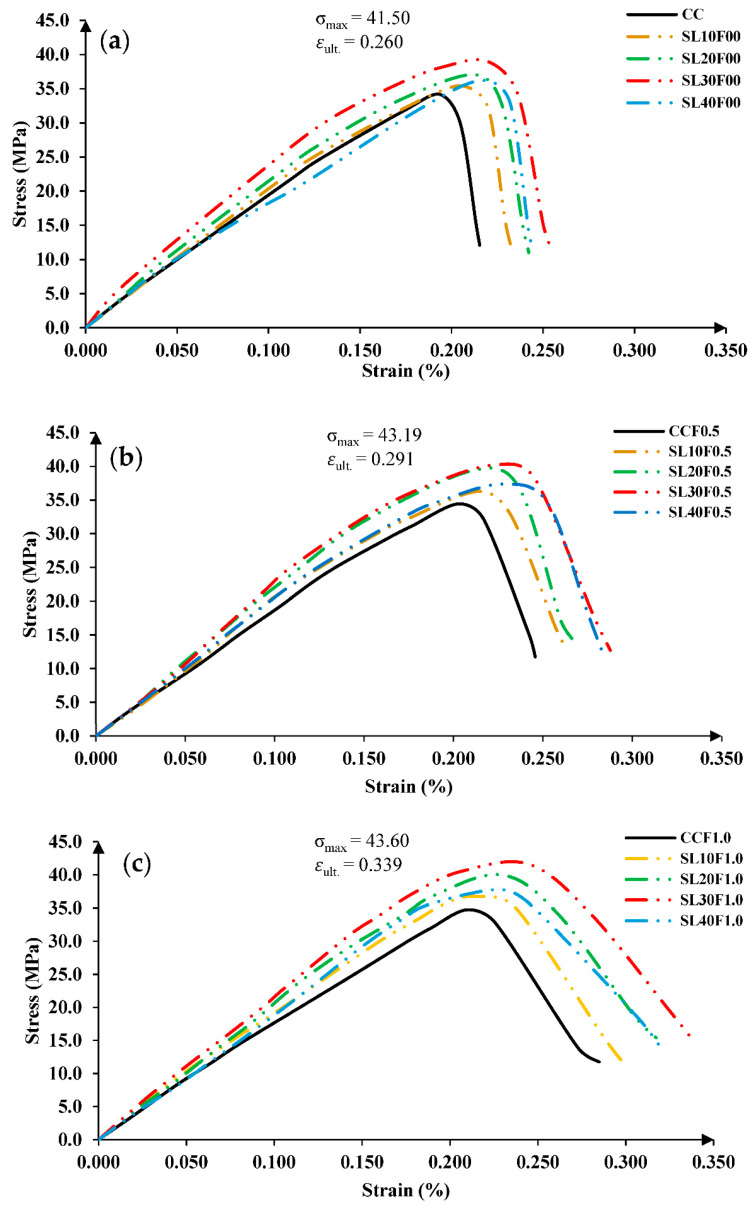
Axial stress–strain curves of all concrete groups (**a**) Slag + 0.0% PP fibers group. (**b**) Slag + 0.5% PP fibers group. (**c**) Slag + 1.0% PP fibers group.

The stress–strain relationship of non-fibrous and fibrous concrete groups is shown in Figure 6a–c, obtained through compression tests of cylindrical specimens with a controlled displacement rate and the use of LVDTs. The effect of natural sand substitution with steel slag is clearly noticeable from the slopes of stress–strain curves. When the sand-replacement material, i.e., steel slag, increases, the slope of the stress–strain curves becomes steeper, representing the increased brittleness and stiffness of mixes along with increased compressive strength and ultimate strains. PP fibers improved the post-cracking behavior of FRSLC, due to which concrete specimens undergo higher ultimate strains and less concrete spalling. 

Furthermore, closely examining the modulus of elasticity results given in Table 7, it is observed that the modulus of elasticity of non-fibrous steel slag concrete is more than that of control concrete.

An increase in modulus of elasticity is at maximum at 30% substitution value. Amplified bonding between the cement paste and aggregate particles due to the high angularity and hardness of the steel slag may be the cause of this increase. With an additional increase in the steel slag substitution, a decrease in the modulus of elasticity was observed. This finding is somewhat compatible with Guo et al. [13] and Dharan and Lal [54], who described that the modulus of elasticity increases till the optimum percentage of the fine aggregate replacement, and decreases afterward. Moreover, the incorporation of polypropylene fibers into steel slag concrete negatively affected the modulus of elasticity, demonstrating the development of ductility in concrete. The experimental results suggest that the modulus of elasticity decreases with the additional quantities of fiber content. This decrease can also be correlated with the mild slope of stress–strain curves in fibrous concrete mixes, as fibers improved the elastic behavior of concrete causing the reduction in the modulus of the elasticity [27,31,41]. Karahan and Atiş [37], Aslani and Nejadi [33], and Das et al. [39] also agreed that the addition of polypropylene fibers does not positively affect the modulus of elasticity.

### 3.3. Flexural Strength

**Figure 7:**
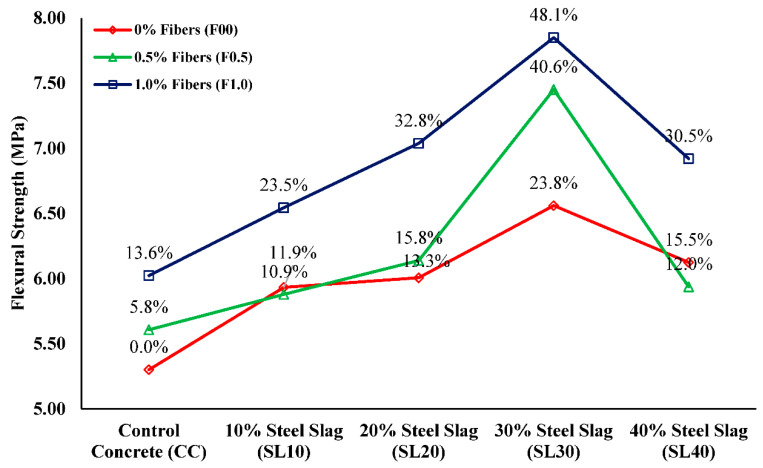
Flexural strength results of all concrete mixes.

**Figure 8:**
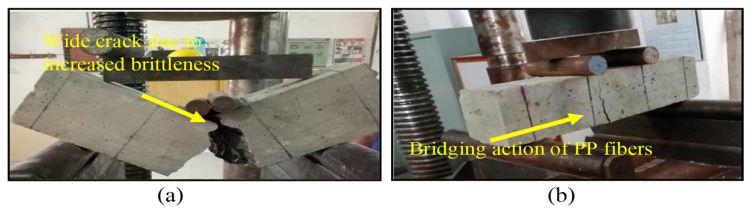
The fracture pattern of concrete specimens in the performance of test for flexural strength. (**a**) In control and steel slag concrete specimens, sudden failure at peak load was seen. (**b**) In fiber-reinforced steel slag concrete specimens, crack width was progressively increased till the ultimate load and both pieces of broken prism remained intact, even at the ultimate load.

Figure 7 demonstrates the variation in flexural strength ($f_{r}$) of mixes duly calculated in terms of modulus of rupture, as per the guidelines of ASTM C78/C78M-15a [55]. Results reflect that steel slag has effectively improved the flexural strength when compared with that of control concrete. In the slag-only concrete group, a considerable increase of 11.9%, 13.3%, 23.8%, and 15.5% was observed upon the replacement of 10%, 20%, 30%, and 40% sand with steel slag, respectively. This increase might be associated with the improved cohesive forces between the aggregate and cement paste due to the superior quality of steel slag in addition to its high angularity. It was also shown that the influence of PP fibers on flexural strength was much more prominent, unlike its influence on compressive strength values. The growth in the fiber content from 0.5% to 1.0% increased flexural strengths. A maximum increase of 48.1% was observed in SL30F1.0 compared with the control concrete. Ostensibly, the combined effect of steel slag and polypropylene fibers leads to the augmentation of flexural strength. For instance, the improvement alone in SL30F00 was 23.8%, which increased by 13.6% and 19.7% upon the addition of 0.5% and 1.0% PP fibers, correspondingly. The addition of PP fibers improved the bond between cement matrix and fibers in the hybrid-fiber-reinforced mix—fibers bridged the micro-cracks and restrained crack growth. The bridging action of fibers is evident from crack widths observed in failure modes shown in Figure 8. Concrete prisms having no fibers were broken into pieces suddenly at peak load with wide cracks, whereas fibrous beams showed very narrow crack widths and did not show sudden failure at peak load. Similar remarks were made by Afroughsabet and Ozbakkaloglu [34] and Mohammadhosseini et al. [56], who discovered that the addition of polypropylene fibers greatly enhances the flexural strength of concrete.

### 3.4. Splitting Tensile Strength

**Figure 9:**
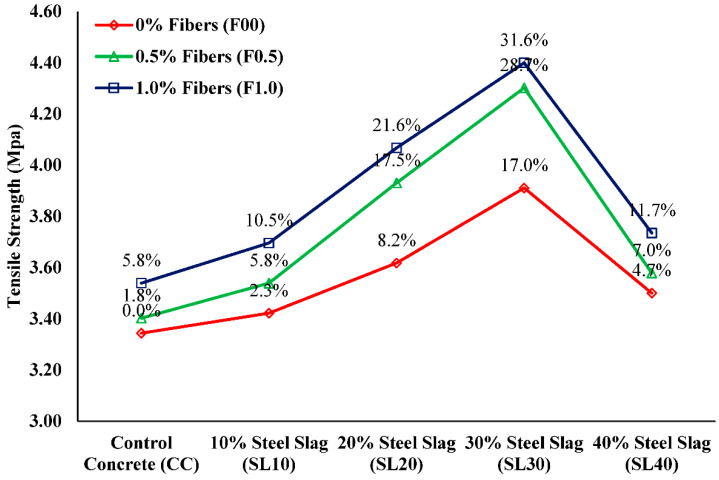
Compare and contrast of split tensile strength test results.

**Figure 10:**
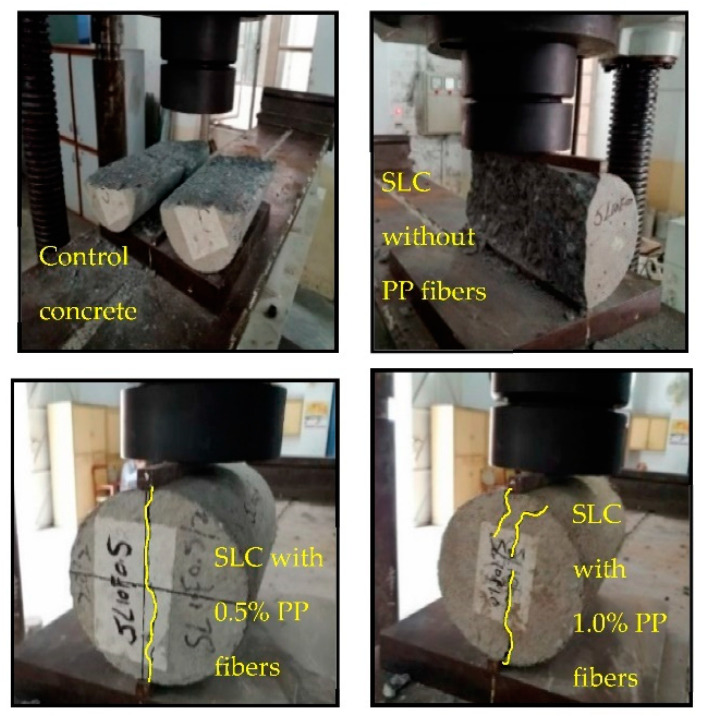
Failure behavior in split tensile strength test.

The variations found in splitting-tensile strength ($f_{t}$) of different mixtures are plotted in Figure 9. Experimental investigations indicate that the split tensile strengths of steel slag concrete specimens are meaningfully better than that of control concrete, and supplementation of PP fibers further alleviates it. Tensile strength in non-fibrous steel slag concrete was 2.3%, 8.2%, 17.0%, and 4.7% higher than control concrete, for 10%, 20%, 30%, and 40% replacement ratios, respectively. The maximum increase of 17% in splitting-tensile strength was attained at 30% replacement of sand, as anticipated due to better bond performance of cement paste caused by the angularity of steel slag [21]. As expected, the rise in the fiber fraction from 0.5% to 1.0% by weight of cement increased tensile strengths. Focusing on the strength results of fibrous steel slag concrete, it can be deduced that the combination of steel slag and polypropylene fibers contributes to the development of concrete tensile strength. The major role in the enhancement of concrete tensile strength was of steel slag than PP fibers. For instance, the maximum rise of tensile strength was 17% in SL30F00 which had an increment of only 10.0% and 12.5% in SL30F0.5 and SL30F1.0 upon the addition of fibers, respectively. When the splitting process occurs, the fibers function as a bridging element that transfers the load from the matrix to the fibers, thus carrying the extra load than non-fibrous concrete specimens which leads to an improvement of split tensile values. Additionally, the observed failure modes in the split tensile test reflect that the non-fibrous specimens were split into two halves, showing the large damage zone. However, FRSLC specimens remained intact, even after peak load, as shown in Figure 10. Although very limited data are available on the combined effect of steel slag and PP fibers, the findings of this research study on tensile strength are somewhat compatible with Mazaheripour et al. [57], who reported that tensile strength of concrete increases with the incorporation of polypropylene fibers to lightweight self-compacting concrete.

### 3.5. Impact Resistance 

The number of hammer blows borne by the concrete disk specimens for the initial cracking (*N_i_*) and ultimate failure (*N_f_*), along with the calculated impact energies using Equations (2) and (3) are given in Table 8. It is obvious from the results that, with the increase in substitution ratio of steel slag with natural sand, final impact energies increase. The improvement in final impact energy was 3.6%, 7.1%, 28.6%, and 14.3% for non-fibrous steel slag concrete mixes (mix No. 2 to 5) compared with control concrete (mix No. 1). This increased impact resistance may be the effect of higher bond strength of aggregate particles with cement past developed due to rough-surfaced steel slag. Research findings also suggest that, with the addition of polypropylene fibers to steel slag concrete, a conclusive increase was seen in the number of blows required for the initial and final cracking of concrete disk specimens. The number of blows required for the first crack appearance was increased by 12%, 48%, 60%, 100%, and 64% for mix No. 6 to 10 having 0.5% PP fibers, and this increase was 16%, 56%, 68%, 108%, and 60% for mix No. 11 to 15 holding 1.0% PP fibers by weight of cement.

**Figure 11:**
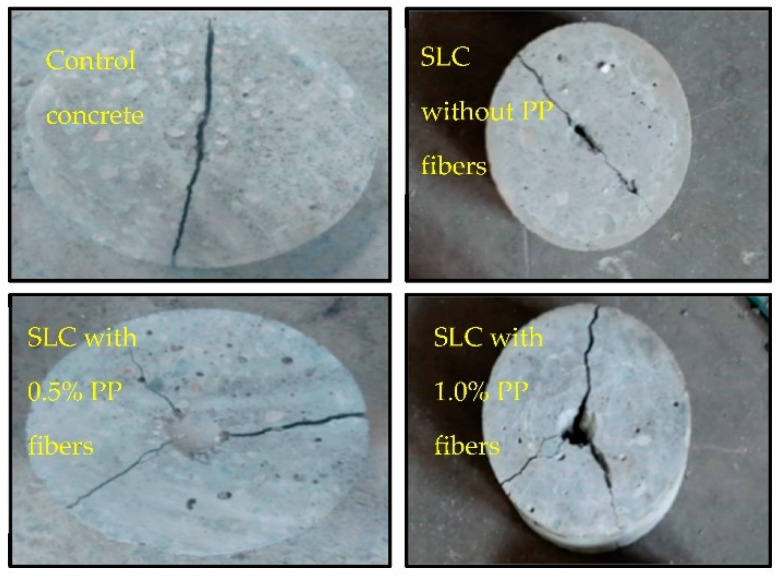
Failed concrete disk specimens in drop weight impact test.

These results also depict the relation between impact energies and fiber dosage. Higher impact energies are found with the increase in PP fiber dosage. For instance, a mixture containing 30% slag without any fibers (mix No. 4) exhibited 732 kN mm of final impact energy which increased to 1261 and 1343 kN mm upon the addition of 0.5% and 1.0% PP fibers (mix No. 9 and 14), respectively. This increase in the impact resistance of fiber-reinforced slag concrete is due to the combined effect of steel slag and PP fibers. The increase in fiber dose raised the fiber density in the mixtures; therefore, more PP fibers were available per unit area to bear the impact forces. Moreover, fibers changed the direction of crack growth and restricted the opening of cracks as can be seen in the failure patterns shown in Figure 11. It can also be noticed that crack shape changed from a single line to a star shape upon the inclusion of fibers to concrete. Although the combined effect of steel slag and PP fibers has not been studied yet under impact forces, these observations on the fiber contents in concrete mixes are somehow similar to the findings of Mohammadhosseini et al. [56], who used waste polypropylene carpet fibers in concrete and found increased impact strength with the increase in fiber content. 

The inclusion of fibers also influences the post-first crack resistance, i.e., $N_{f}$ −$\;\;N_{i}$, see Table 8. The increase in post-crack performance is perceptible in fibrous concrete mixes having steel slag content between 20% and 40%, which means that a greater number of blows are required for the final failure of the concrete specimen after the appearance of the first crack, indicating the ductile behavior of fiber-reinforced slag concrete due to the bridging action of PP fibers.

### 3.6. Water Absorption and Unit Weight

**Figure 12:**
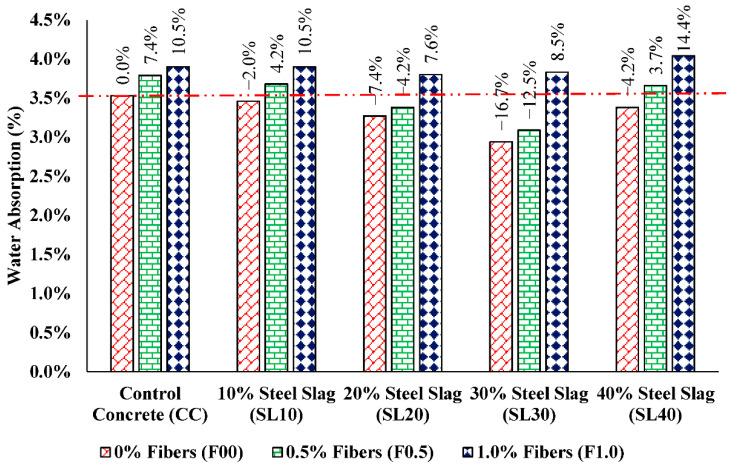
Comparison of water absorption results for all mixes.

Water absorption is an important parameter to comment on the durability of concrete. It is simply the measure of concrete resistance in aggressive environments because it indirectly reflects the perviousness of concrete. The results of water absorption are produced in Figure 12. Non-fibrous steel slag concrete specimens showed a decrement in water absorption upon the increase in steel slag quantity till the optimum percentage of natural sand replacement when compared with control concrete specimens. The maximum drop observed in water absorption was 17% in a mix having 30% steel slag without PP fibers (SL30F00). The reason behind this reduction in water absorption is less porosity of the concrete mixes upon the incorporation of steel slag which made the concrete denser. Increased compressive strength also validates this. Singh and Siddique [58] studied the durability characteristics of self-compacting concrete holding iron slag as fine aggregate material and reported that steel slag concrete had less water absorption than normal concrete. However, with the addition of PP fibers, the water absorption of steel slag concrete is found to be slightly increased. This might be due to the interlinkage of pores upon the addition of fibers and a non-homogeneous mix. The results of this research are comparable with the findings of Karahan and Atiş [37], who studied the effect of polypropylene fiber on concrete properties and found increased water absorption along with an improvement in compressive strength. It is also worth noting that water absorption of all concrete mixes is not more than 5% which generally represents good quality concrete [34].

**Figure 13:**
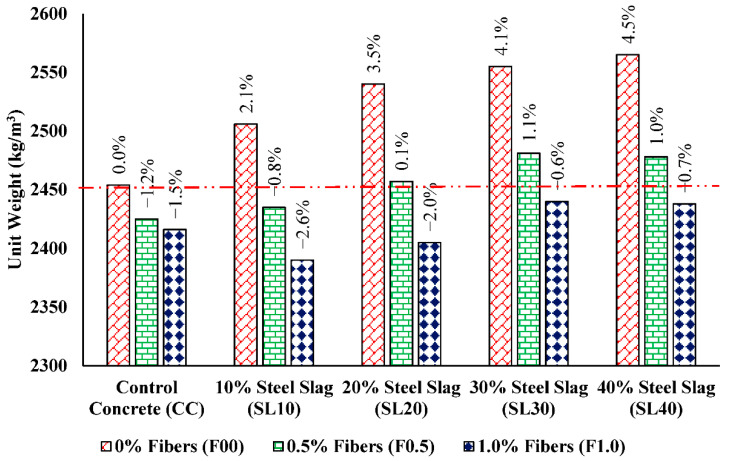
Comparison of dry unit weight results for all mixes.

The dry unit weight of each mix was also checked, and the resulting values are produced in Figure 13. It is perceived that with the increase in the substitution ratio of slag, the dry density of concrete mixes increased because the specific gravity of slag is greater than that of sand. However, the concrete in all cases is considered to be normal-density concrete when correlated with the guidelines stated in ACI 304.3 “Heavyweight Concrete” [59]. The addition of PP fibers slightly reduces the density of concrete, lesser specific gravity of the fibers supports these results; when the percentage of fibers increased, it replaced other concrete ingredients to achieve the same volume of the cylinder being used for testing. Karahan and Atiş [37] found that the mixes reinforced with PP fibers had slightly lower dry unit weights than non-fibrous concrete specimens.

### 3.7. Microstructural Analysis 

**Figure 14:**
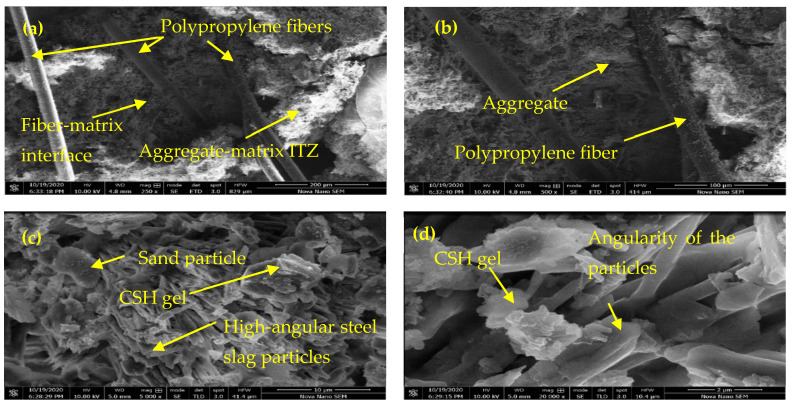
Microstructure analysis of fiber-reinforced slag concrete (**a**) bridging action of PP fibers. (**b**) enlarged view of PP fibers. (**c**) effective coating of cement particles around fine aggregates. (**d**) high-angular steel slag.

Microstructural analysis of slag concrete is performed with the help of scanning electron microscopy (SEM) using the Everhart–Thornley detector (ETD) and through-lens detector (TLD) to examine the integral structure and combined effects of steel slag and polypropylene fibers. The SEM images obtained are produced in Figure 14a–d. Figure 14a reveals the bridging action offered by PP fibers as one end of the fiber is rooted in the concrete matrix. These bridged PP fibers, in combination with the steel slag, joined the micro cracks firmly and did not allow the cracks to widen. The enlarged view (Figure 14b) also depicts the existence of a strong interfacial transition zone (ITZ) around the PP fiber between the coarse aggregate and the mortar matrix, which consequently has enhanced the mechanical parameters of fiber-reinforced slag concrete more than CC by restricting and delaying the generation of the cracks. On the other hand, the high angularity of the steel slag particles and effective coating of cement particles around them is shown in Figure 14c,d, which has also improved the bond strengths, leading to improved characteristics of the slag concrete. The majority of the test results are obtained by mechanical means, even though findings obtained through SEM analysis validate some of the whys and wherefores identified from the results and produced in earlier sections. It also encourages our findings deduced by mechanical means.

## 4. Analytical Analyses 

### 4.1. Regression Analysis 

**Figure 15:**
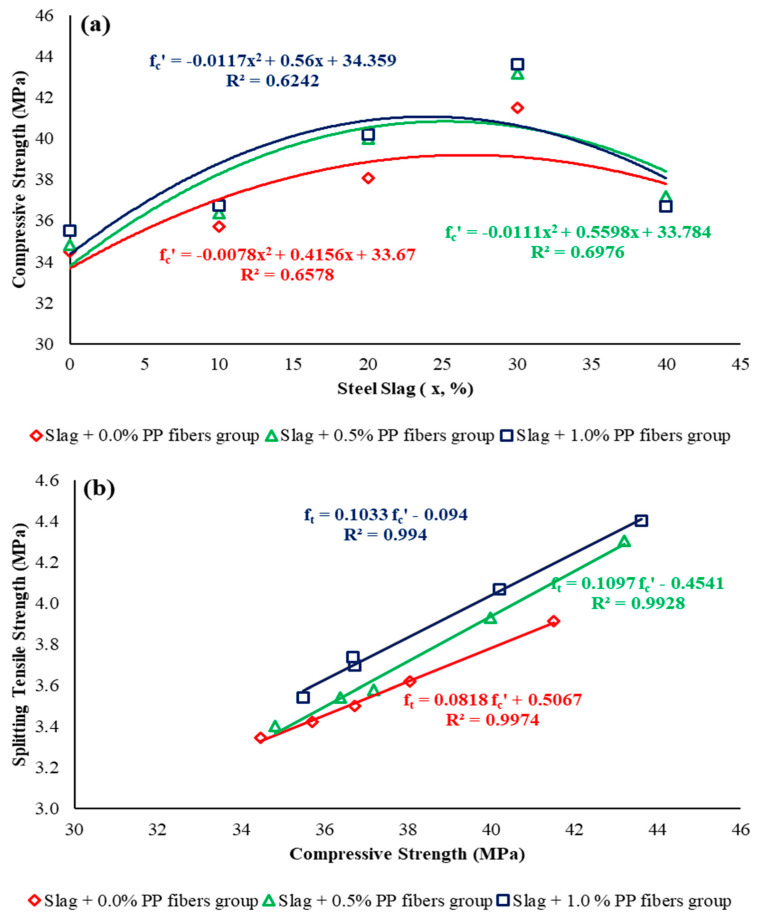

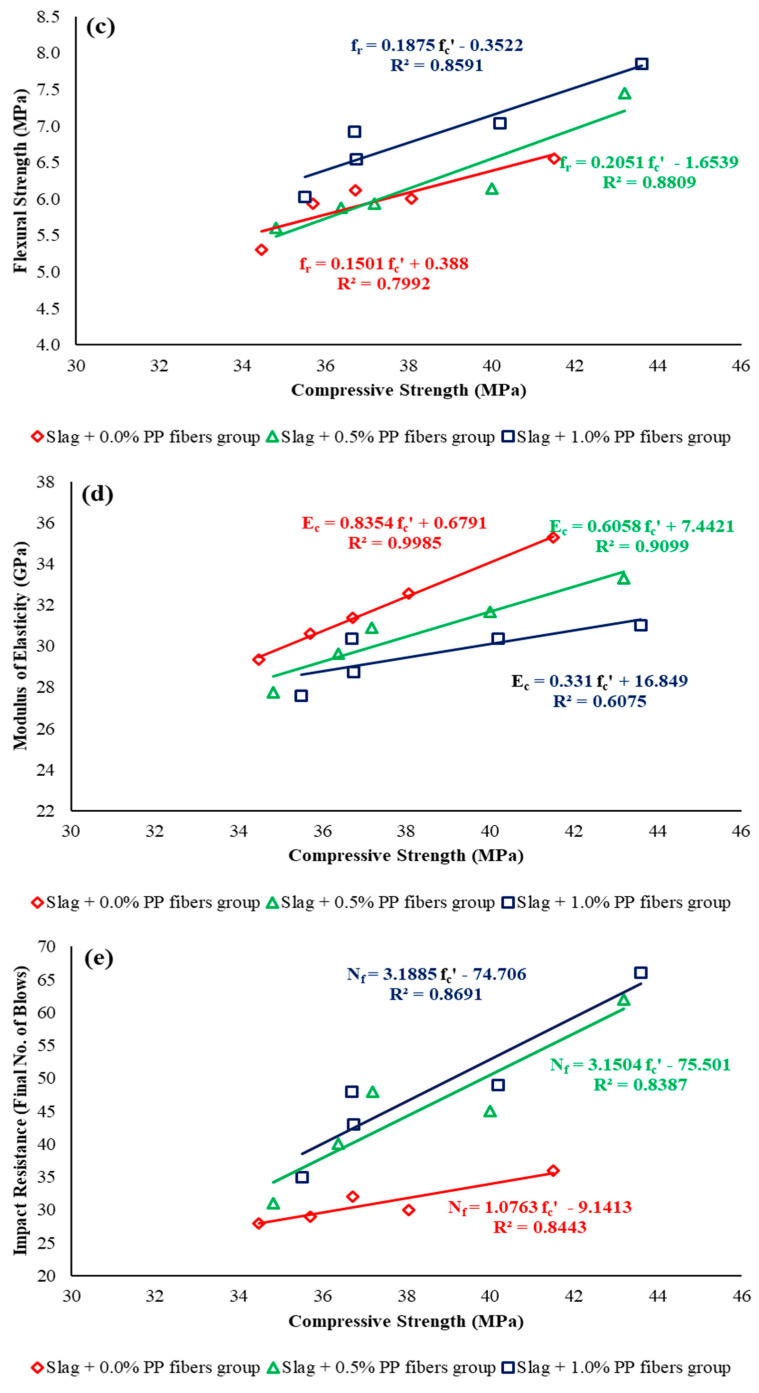

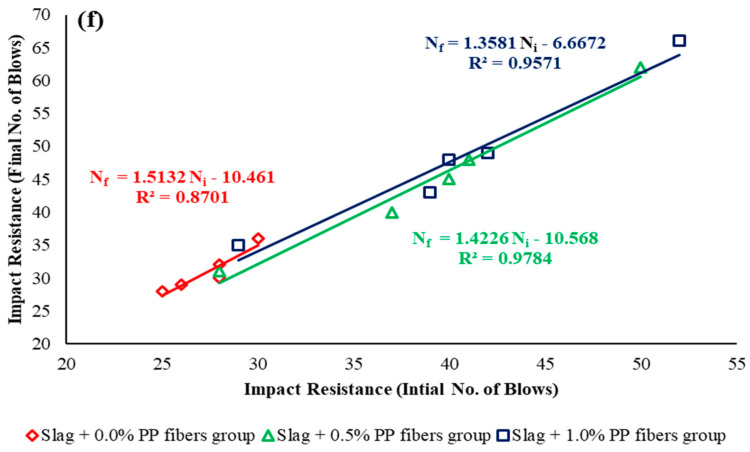
Relationship between (**a**) steel slag (%) and compressive strength; (**b**) compressive strength and splitting tensile strength; (**c**) compressive strength and flexural strength; (**d**) compressive strength and modulus of elasticity; (**e**) compressive strength and impact resistance (final No. of blows); (**f**) impact resistance (initial No. of blows vs. final No. of blows).

The experimental results are assessed analytically in this section using polynomial and linear regressions (Figure 15) to elaborate on the effects of natural sand replacement with steel slag and the addition of PP fibers to the mechanical and impact behaviors of the concrete. The empirical equations obtained for the relationship between tested variables, along with R2 (coefficient of correlation for regression models), are shown separately in each graph. In statistics, R2 ranges from 0 to 1, where 0 indicates the least data match and 1 represents the perfect regression relationship. It is evident that compressive strength follows the polynomial trend for increasing replacement levels of natural sand with steel slag (Figure 15). The maximum compressive strength was obtained at a 30% substitution ratio of steel slag and decreased with the further increase in substitution ratio for all three concrete groups, with the R2 value ranging from 0.6242 to 0.6976. The equations developed are shown in each graph in Figure 15. As an illustration, the relationships shown in Figure 15a, “x”, denote the steel slag percentage, and ${f_{c}}^{\prime }$ represents the compressive strength of the mixes. Nevertheless, a linear relationship is observed when the compressive strength is compared with the other strength parameters. The results also reveal that the combined use of PP fibers and slag in concrete have substantially augmented the tensile and flexural strengths (Figure 15b,c). The use of PP fibers in slag concrete has attained a higher increase in flexural strength with rising compressive strength than tensile strength. However, with the addition of PP fibers, a higher R2 value is obtained for tensile strength in contrast to flexural strength. The addition of PP fibers to the slag concrete reduces *Ec* and R2, due to introduced elastic behavior because of the addition of fibers, as shown in Figure 15d [41]. The relationship between the compressive strength and impact resistance corresponding to the final no. of blows, shown in Figure 15e, reflects that the Slag + 0.0% PP fibers group possesses low impact resistance compared with the fibrous groups. The relation between initial and final number of blows reflects that the addition of fibers enhances the R2 value from 0.8701 to 0.9784 upon the addition of 0.5% PP fibers (Figure 15f). Additionally, the mathematical models developed in this section are only applicable for the tested concrete specimens with constituent material properties, similar to the properties defined in Section 2.1.

**Figure 16:**
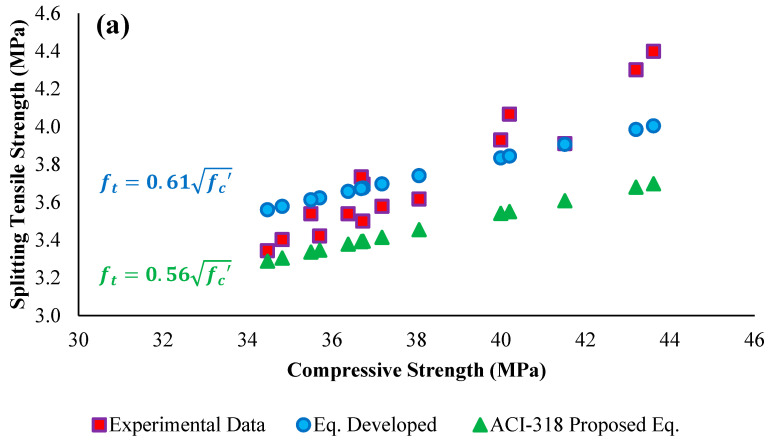

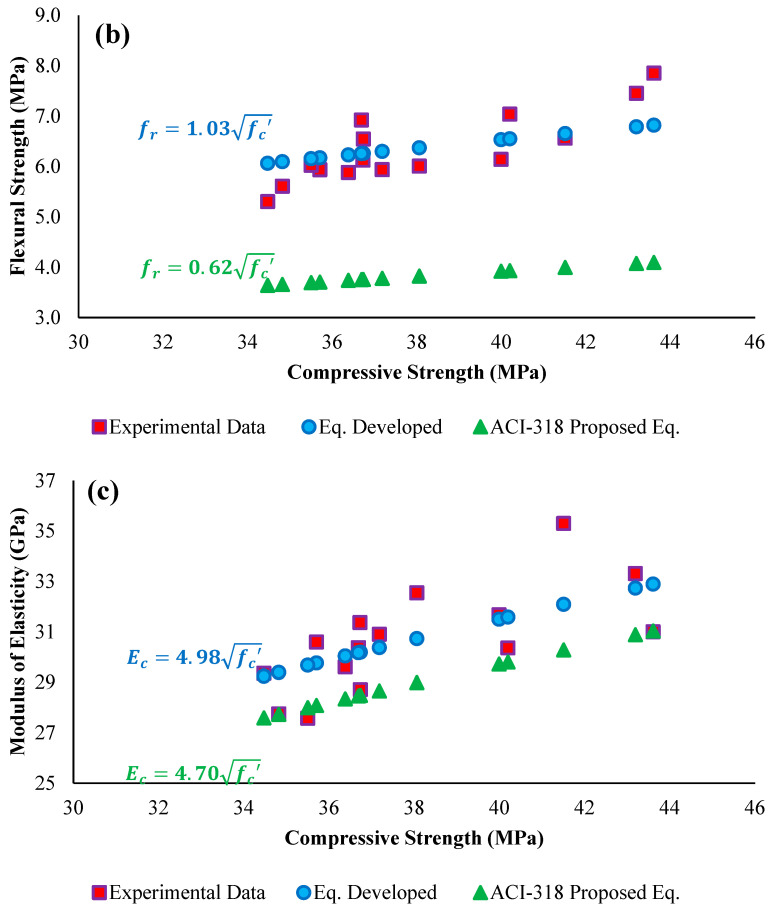
Comparison of experimental data with ACI proposed models and developed equations between (**a**) compressive strength and tensile strength; (**b**) compressive strength and flexural strength; (**c**) compressive strength and modulus of elasticity.

The experimental data have also been compared with ACI-318M-14 [60] strength-predicting models for the relationships between compressive strength and other mechanical parameters, and the results are shown in Figure 16a–c. It is evident from the obtained results that the experimental data are better compared with the ACI-expected data.

### 4.2. Probability Analysis 

This section discusses the probability distribution of the mechanical and impact behaviors of all studied concrete mixes. The use of waste steel slag leads to greater uncertainties in experimental results because of its high angularity and irregular-shaped particles along with varied sizes. However, the extent of these uncertainties can be well realized through statistical analyses. Therefore, the probability distribution of various concrete properties is carried out in this research with the help of the computer program OriginLab. Normality of experimental data has been carried out by the calculation of p-values through a goodness-of-fit test by the Shapiro–Wilk method, and the resulting *p*-values are given in Table 9.

The *p*-values are calculated by assuming the normal distribution with a significance level of 0.05. Correspondingly, any *p*-value which is equal to or lower than 0.05 means that the assumption is not satisfied, and the data do not fit the normal distribution with 95% confidence [61]. Comparing the calculated *p*-values given in Table 9 with this assumption indicates that no value is less than 0.05, ultimately suggesting that mechanical and impact properties obtained in this research study are normally distributed, regardless of the combined use of waste steel slag and polypropylene fibers.

## 5. Conclusions

An experimental investigation was conducted to explore the influence of PP fibers on the static and impact performance of the concrete having steel slag as sand-replacement material. The experimental investigations showed that fiber-reinforced slag concrete (FRSLC) exhibited better mechanical and microstructural properties than control concrete. The conclusions drawn from the study are summarized as follows:

- Mechanical properties of slag concrete increases with the increase in volumetric substitution ratio of steel slag up to 30% and then reduces. Hence, the optimum content of natural sand replacement with the steel slag is 30%.
- Polypropylene fibers have a small effect on compressive strength as compared with the improvements in other mechanical parameters of fiber-reinforced steel slag concrete specimens. Fiber addition of 1.0% to the weight of the cement is discovered to be the optimum dosage for all strength parameters.
- Sand replacement with the steel slag enhances the stiffness and brittleness of concrete. The static modulus of the best-declared mix without fibers (i.e., SL30F00) is 20% higher than the control concrete. The addition of PP fibers improves the post-first crack performance and ductility of the concrete, slightly reducing the modulus of its elasticity to 6% in SL30F1.0.
- The cumulative use of steel slag and PP fibers increases the compressive, flexural, and split tensile strength for the optimized percentage replacement of sand without fibers by 26%, 48%, and 32% with regard to the referenced mix.
- The combination of steel slag and PP fibers meaningfully improved the drop weight impact resistance of steel slag concrete. The maximum improvement of 108% and 164% in initial and final impact energies are reported, respectively in SL30F1.0 when compared with those of control concrete.
- Water absorption reduces, and dry density increases with the growth in steel slag content. The increases of 2.1%, 3.5%, 4.1%, and 4.5% in dry density are observed upon 10%, 20%, 30%, and 40% steel slag incorporation with respect to control concrete, and these increases are within the limits of normal density concrete stated in ACI 304.3-12 “Heavyweight Concrete”.
- The microstructural studies validate the bridging action of PP fibers and the high angularity of steel slag as a major reason for the enhancement of mechanical parameters of FRSLC.
- The analytical studies reveal that mechanical and impact behaviors of FRSLC mixtures followed a normal distribution and are the best fit for this study. Moreover, the expressions developed for the FRSLC models are better than the ACI-318M-14 models.

Based on the findings obtained and analyzed behaviors, FRSLC is anticipated to be successful in reducing the unfavorable effects of the steel slag by utilizing its large quantities in the concrete. Moreover, it also plays a significant role in evolving the energy-absorption capabilities of concrete. Among the investigated types of the FRSLC, concrete mix with 30% steel slag as a sand-replacement material and the addition of 1.0% polypropylene fibers is the most effective because it depicts the highest improvement in most of the investigated parameters, compared with other studied mixes. However, this study has been conducted for low-velocity impact loads; therefore, a similar study under high-velocity impact is recommended to be conducted on a prototype model of structures in order to proceed toward its practical applications.

## Figures and Tables

**Table 1:** Chemical and physical properties of cement and steel slag used.

Chemical Composition		
Oxides	Cement (%)	Steel Slag (%)
SiO_2_	20.41	8.92
Al_2_O_3_	5.14	3.78
Fe_2_O_3_	3.13	83.63
CaO	63.32	0.24
MgO	2.5	0.38
MnO	-	-
SO_3_	2.75	2.27
K_2_O	0.65	-
Na_2_O	0.16	-
TiO_2_	-	-
Cl	0.0016	-
IR	0.55	-
LOI	2.02	1.61
C_3_A	8.33	-
AE	0.6	-
Moisture Content	-	0.54
**Physical properties**		
Specific gravity	3.15	3.12
Initial setting time	46 min	-
Final setting time	215 min	-
Lechatiler’s expansion	0.5 mm	-
Fineness by Blaine	336 m^2^ kg^−1^	-
Standard consistency	27%	-
3 days, compressive strength	40.6 MPa	-
28 days, compressive strength	59 MPa	-

**Table 2:** Physical properties of natural sand and steel slag.

No.	Description	Natural Sand	Steel Slag
1	Specific gravity (OD)	2.73	3.12
2	Specific gravity (SSD)	2.76	3.15
3	Apparent specific gravity	2.81	3.24
4	Water absorption (%)	1.02	1.20
5	Fineness modulus	2.52	3.00

**Table 3:** Physical properties of natural coarse aggregates.

No.	Description	Value
1	Specific gravity (OD)	2.78
2	Specific gravity (SSD)	2.80
3	Apparent specific gravity	2.84
4	Water absorption (%)	0.75
5	Aggregate crushing value (%)	20.2
6	Aggregate impact value (%)	14.83
7	Loss Angeles abrasion value (%)	14.06
8	Flakiness index (%)	23.16
9	Elongation index (%)	26.69
10	Sodium sulphate soundness (%)	1.05

**Table 4:** Properties of polypropylene fibers.

No.	Description	Value
1	Color	Translucent white
2	Nominal cross-sectional area (mm^2^)	0.75
3	Melting point (°C)	150–170
4	Density (gm/cm^3^)	0.88–0.92
5	Fiber length (mm)	12
6	Fiber diameter (micron)	18
7	Elongation at yield (%)	24.4
8	Water absorption (%)	0
9	Acid/Alkali resistance	High
10	Tensile strength at yield (MPa)	320–400
11	Young’s modulus (MPa)	3500–3900

**Table 5:** Concrete mix proportions.

Mix No.	Mix Designation	Cement (kg/m^3^)	Water (kg/m^3^)	Coarse Aggregate (kg/m^3^)	Natural Sand (kg/m^3^)	STEEL SLAG		Admixture (kg/m^3^)	Fiber Content (%)
						Ratio	(kg/m^3^)		
**Slag + 0% PP fibers group**									
1	CC	410	198	1040	750	0%	0	0	0
2	SL10F00	410	198	1040	675	10%	86	0	0
3	SL20F00	410	198	1040	600	20%	172	0	0
4	SL30F00	410	198	1040	525	30%	257	0	0
5	SL40F00	410	198	1040	450	40%	343	0	0
**Slag + 0.5% PP fibers group**									
6	CCF0.5	410	198	1040	750	0%	0	3.69	0.5
7	SL10F0.5	410	198	1040	675	10%	86	3.69	0.5
8	SL20F0.5	410	198	1040	600	20%	172	3.69	0.5
9	SL30F0.5	410	198	1040	525	30%	257	3.72	0.5
10	SL40F0.5	410	198	1040	450	40%	343	3.75	0.5
**Slag + 1% PP fibers group**									
11	CCF1.0	410	198	1040	750	0%	0	4.10	1.0
12	SL10F1.0	410	198	1040	675	10%	86	4.10	1.0
13	SL20F1.0	410	198	1040	600	20%	172	4.10	1.0
14	SL30F1.0	410	198	1040	525	30%	257	4.15	1.0
15	SL40F1.0	410	198	1040	450	40%	343	4.15	1.0

Note: SL = Steel slag, CC = Control Concrete, F = Fibers. Steel slag substitutions are made by volume. Fiber percentage is calculated by weight of cement mass. SL30F1.0 mean, that this mix has 30% sand replaced with steel slag by volume of sand and 1.0% PP fibers added by weight of cement.

**Table 6:** Specimen dimensions for different tests.

No.	Test Name	Test Standard	Specimen Dimensions
1	Compressive strength	ASTM C39/C39M-15a	150 mm diameter and 300 mm height
2	Flexural strength	ASTM C78/C78M-15a	100 mm by 100 mm cross-section and 400 mm length
3	Split tensile strength	ASTM C496/C496M-11	150 mm diameter and 300 mm height
4	Drop weight impact strength	ACI 544.2R-89	150 mm diameter and 63.5 mm height
5	Water absorption	ASTM C642-13	150 mm diameter and 300 mm height
6	Unit weight	ASTM C642-13	150 mm diameter and 300 mm height
7	Modulus of elasticity	ASTM C469/C469M-14	150 mm diameter and 300 mm height

**Table 7:** Summary of experimental results of all concrete mixes.

Mix No.	Mix Designation	Compressive Strength (MPa)	Splitting Tensile Strength (MPa)	Flexural Strength (MPa)	Modulus of Elasticity (MPa)	Unit Weight (kg/m^3^)	Water Absorption (%)
**Slag + 0% PP fibers group**							
1	CC	34.472	3.344	5.301	29.361	2454	3.53
2	SL10F00	35.706	3.422	5.932	30.597	2506	3.46
3	SL20F00	38.060	3.617	6.006	32.547	2540	3.27
4	SL30F00	41.508	3.911	6.560	35.299	2555	2.94
5	SL40F00	36.726	3.500	6.124	31.370	2565	3.38
**Slag + 0.5% PP fibers group**							
6	CCF0.5	34.820	3.402	5.607	27.753	2425	3.79
7	SL10F0.5	36.381	3.539	5.879	29.614	2435	3.68
8	SL20F0.5	39.992	3.930	6.139	31.677	2457	3.38
9	SL30F0.5	43.192	4.302	7.451	33.313	2481	3.09
10	SL40F0.5	37.181	3.578	5.937	30.902	2478	3.66
**Slag + 1% PP fibers group**							
11	CCF1.0	35.500	3.539	6.023	27.582	2416	3.90
12	SL10F1.0	36.735	3.696	6.544	28.717	2390	3.90
13	SL20F1.0	40.200	4.067	7.038	30.364	2405	3.80
14	SL30F1.0	43.607	4.399	7.850	31.003	2440	3.83
15	SL40F1.0	36.691	3.735	6.920	30.371	2438	4.04

Note: (1) All values are corresponding to the 28-day test results. (2) Underlined results are at maximum in each concrete group. (3) The values are the average of a minimum of three specimens.

**Table 8:** Drop weight impact test results.

Mix No.	Mix Designation	Impact Resistance (No. of Blows)		*N_f_* − *N_i_*	Impact Energy (kN-mm)	
		First Visible Crack	Failure of Specimen		Initial	Final
		*N_i_*	*N_f_*		*E_i_*	*E_f_*
**Slag + 0.0% PP fibers group**						
1	Control Concrete	25	28	3	509	570
2	SL10F00	26	29	3	529	590
3	SL20F00	28	30	2	570	610
4	SL30F00	30	36	6	610	732
5	SL40F00	28	32	4	570	651
**Slag + 0.5% PP fibers group**						
6	CCF0.5	28	31	3	570	631
7	SL10F0.5	37	40	3	753	814
8	SL20F0.5	40	45	5	814	916
9	SL30F0.5	50	62	12	1017	1261
10	SL40F0.5	41	48	7	834	977
**Slag + 1.0% PP fibers group**						
11	CCF1.0	29	35	6	590	712
12	SL10F1.0	39	43	4	794	875
13	SL20F1.0	42	49	7	855	997
14	SL30F1.0	52	66	14	1058	1343
15	SL40F1.0	40	48	8	814	977

Note: (1) All values are corresponding to 28-day test results. (2) Underlined results are maximum in each concrete group. (3) The results are the average of a minimum of three specimens.

**Table 9:** *p*-values obtained through the Shapiro–Wilk method for the normality of data.

No.	Test Name	Slag + 0% PP Fibers Group	Slag + 0.5% PP Fibers Group	Slag + 1% PP Fibers Group
1	Compressive strength	0.691	0.700	0.294
2	Flexural strength	0.950	0.071	0.934
3	Split tensile strength	0.522	0.416	0.526
4	Modulus of elasticity	0.791	0.994	0.374
5	Initial impact resistance	0.758	0.867	0.753
6	Final impact resistance	0.482	0.928	0.647
7	Post first crack impact resistance (*N_f_* − *N_i_*)	0.492	0.235	0.382

## Data Availability

The data supporting the findings of this study are available from the corresponding author on request.
